# Recent Advancements in Rubber Composites for Physical Activity Monitoring Sensors: A Critical Review

**DOI:** 10.3390/polym17081085

**Published:** 2025-04-17

**Authors:** Vineet Kumar, Md Najib Alam, Gaurav Manik, Sang-Shin Park

**Affiliations:** 1 School of Mechanical Engineering, Yeungnam University, 280 Daehak-Ro, Gyeongsan 38541, Gyeongbuk, Republic of Korea; vineetfri@gmail.com (V.K.); mdnajib.alam3@gmail.com (M.N.A.); 2 Department of Polymer and Process Engineering, Indian Institute of Technology Roorkee, Saharanpur Campus, Saharanpur 247001, Uttar Pradesh, India; gaurav.manik@pe.iitr.ac.in

**Keywords:** graphene, carbon nanotubes, sensors, sports monitoring, durability

## Abstract

This review provides the latest insight (2020 to 2025) for composite-based physical activity monitoring sensors. These composite materials are based on carbon-reinforced silicone rubber. These composites feature the use of composite materials, thereby allowing the creation of new generation non-invasive sensors for monitoring of sports activity. These physical sports activities include running, cycling, or swimming. The review describes a brief overview of carbon nanomaterials and silicone rubber-based composites. Then, the prospects of such sensors in terms of mechanical and electrical properties are described. Here, a special focus on electrical properties like resistance change, response time, and gauge factor are reported. Finally, the review reports a brief overview of the industrial uses of these sensors. Some aspects are sports activities like boxing or physical activities like walking, squatting, or running. Lastly, the main aspect of fracture toughness for obtaining high sensor durability is reviewed. Finally, the key challenges in material stability, scalability, and integration of multifunctional aspects of these composite sensors are addressed. Moreover, the future research prospects are described for these composite-based sensors, along with their advantages and limitations.

## 1. Introduction

The physical activity monitoring sensors are a hot research topic due to their ability to track and measure various physical activities. These physical activities are running, cycling, or swimming in living beings [1,2]. These sensors have various gadgets like temperature sensors, global positioning systems (GPS), or strain gauges. Here, the temperature sensors provide insights about external weather conditions during sports activity. The GPS sensors use satellite signals to obtain geographical locations [3]. They are often used to track remote outdoor sports activities during hiking or cycling. Moreover, the strain gauges assist in measuring the change in strain or mechanical deformation of wearable and flexible materials. For example, smart clothing monitoring or intense sports activity [4,5]. In some cases, the biosensors are helpful in studying the electric impedance of biological systems. These systems can provide sweating, hydration levels, and muscle fatigue or injuries during sporting. Finally, the pressure sensors help to measure the change in air pressure during hiking or other change in altitude during climbing [6]. Therefore, these composite based sensors can be useful by configuring various monitoring activities that test fitness goals, and other optimized training routines [7].

**Scheme 1:**
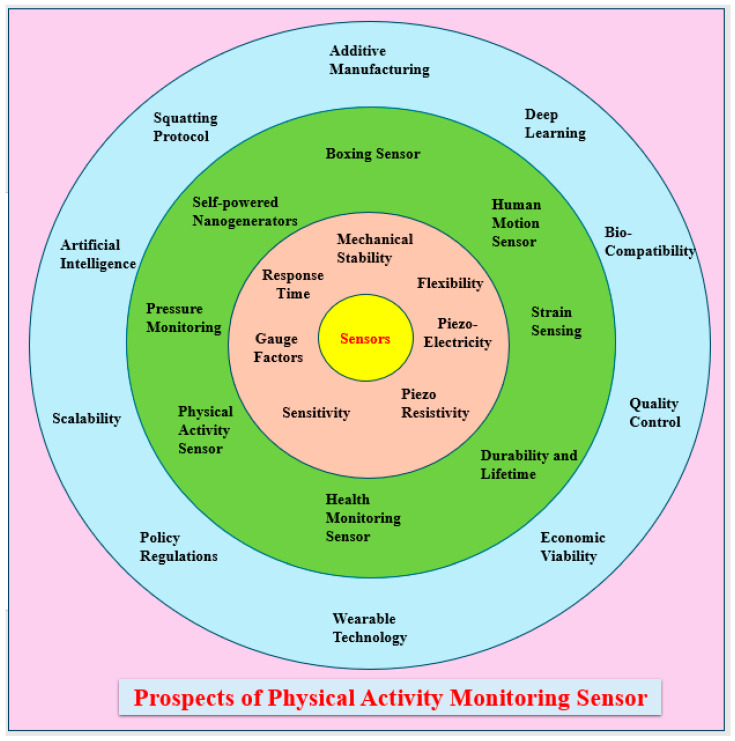
Overview of present work. Inner ring summarizes properties of explored sensors, middle ring explores their applications, and outermost ring explores the prospects of this work for different commercial uses.

These physical activity sensors have numerous advantages for living beings, including humans, aquatic animals, and pulse monitoring in plants [8,9,10]. For example, the composite-based sensors provide quantitative data about the sports activity involved. Here, the athlete wears the sensor to track progress and make informative decisions about their fitness and health. These wearable sensors offer real-time monitoring, thereby allowing the users to receive immediate feedback and control the biological fatigue. Overall, these sensors help in studying the long-term monitoring of sports activity and the well-being of the athlete [11,12]. It, therefore, provides valuable information on exercise monitoring and helps in identifying risk factors. However, despite the multifunctional uses of these sensors, they have limitations as well. Some of the major limitations are that these sensors have issues like accuracy and reliability. These factors depend on various factors like sensor type, calibration, or environmental factors [13]. Another limitation includes their comfort, breathability, and ability to wear for long time. This causes discomfort to wear, or skin irritation, and finally, the final cost of the sensor is an issue [14]. Therefore, these limitations need further attention in terms of their commercialization. The overview of the present work is summarized in Scheme 1 below.

Despite various configurations of physical activity sensors, their performance is strongly influenced by the nature of materials used. These sensors are widely fabricated through stretchable and flexible elastomers like silicone rubber [15]. These silicone rubbers are useful due to their ease of processing and ease of vulcanization. Silicone rubber is a versatile material that is widely used as a synthetic elastomer. It consists of silicon, carbon, hydrogen, and oxygen. Due to its exceptional properties, it is widely used in various industrial applications [16]. Based on vulcanizations, there can be room-temperature, low-temperature, and high-temperature silicone rubbers [17,18]. Other flexible substrates like polyimide can withstand repeated bending and stretching without compromising sensor performance [19]. The key features of silicone rubber are thermal stability, flexibility, elasticity, chemical resistance, and electrical insulation [20].

These features can be understood more efficiently. For example, the silicone rubber is stable under a large temperature range, that is, from −60 °C to 250 °C. Thus, it can withstand a large range of temperatures without degrading. This also makes it useful for prolonged exposure to heat [21]. Silicone rubber has excellent flexibility in a large range of temperatures. These aspects make it useful for flexible substrates in stretchable sensors. Moreover, it has high elastic recovery, thereby maintaining its shape under mechanical loads. The silicone rubber is also highly stable to chemicals, including water, oils, and organic solvents. The used solvents make them useful for challenging environments [22]. Moreover, medical-grade silicone rubber can be useful for various implant medical applications. Finally, silicone rubber has excellent electrical insulating properties, making it useful for electrically insulating coatings and electrical cables [23]. Overall, silicone rubber has high weather and UV resistance, making it useful for various indoor applications.

There are various applications for silicone rubber, such as automotive, medical and healthcare, electronics, and construction applications [24]. For example, silicone rubber is extensively used in automobile applications like gaskets, seals, hoses, and thermal insulations. For electronic purposes, it can be helpful for electronic insulators such as cable components [25]. Finally, silicone rubber is useful for making baby products and personal care items due to its non-toxic nature. Overall, the unique properties of silicone rubber make it an indispensable material in modern manufacturing and technology [26]. With continuous research on silicone rubber, it will likely find even more applications, further demonstrating its versatility. Despite this, the sensor should have an electrode with high electrical conductivity and thermal stability. For this, inorganic nanomaterials like carbon black, graphene, carbon nanotubes, carbon nanofibers, and fullerene are often added [27,28,29]. They act as a reinforcing filler material in the rubber matrix and are described in detail in the coming sections.

[i] Carbon black: Carbon black is a fine black product obtained from the incomplete combustion of heavy petroleum. It is widely acknowledged that carbon black can reinforce rubber matrix and has thus been used as filler for more than a century [30]. It is widely used in tires and strain sensors due to its high reinforcing and electrical properties. The main properties of carbon black are small particle size, high surface area, and favorable surface chemistry. These properties can be detailed as the particle size of carbon black ranges from 10 to 500 nm [31]. The smaller the particle size, the better the reinforcement offered to the rubber matrix. Similarly, the larger surface area provides better reinforcing properties by improving the filler–rubber interfacial area. Similarly, the higher structure carbon blacks have more branches and voids that result in improved properties like reinforcing properties and electrical and thermal properties [32]. Moreover, the oxygen-carrying functional groups simulate its surface activity. This activity is enhanced by interfacial interactions between filler and rubber matrix. Carbon black has various applications as a reinforcing agent in tires, strain sensors, and automotive components like vibration-damping components [33,34]. Overall, carbon black plays a crucial role in enhancing the reinforcing properties of the rubber matrix, making it useful for various applications like automotive or strain sensing.
[ii] Graphene: Graphene is a single layer of sp^2^ hybridized carbon atoms arranged in a two-dimensional honeycomb lattice. Graphene is also known as the mother of all carbon allotropic forms like carbon nanotube and graphite [35]. It is well known for its wonderful mechanical, electrical, and thermal properties. These unique properties make it an ideal filler candidate to make the rubber matrix useful for strain-sensing applications [36]. For example, the addition of graphene in rubber matrix composites makes them ultra-strong and able to withstand high mechanical deformation. Their high electrical properties also make them useful for superior electrical conductivity. This is due to high electron mobility of graphene [37]. For sensor applications, the piezoresistive effect and short response time of graphene enhance the strain sensitivity. This allows them to detect even minor stimuli. Graphene provides fatigue resistance and promotes repeated bending and stretching without performance degradation [38]. Moreover, graphene also reduces noise and improves the accuracy of strain measurements, thereby making the strain sensor robust and exhibit high performance. This noise can be mechanical, electrical, material-inherent, or contact noise. Here, the mechanical noise can be sourced from mechanical vibrations and shocks, thereby leading to unstable energy outputs. Then, electrical noise is due to internal parasitic components in the sensor, and it distorts output signal integrity. Then, the material-inherent noise is sourced from non-uniform filler dispersion for composite-based sensors. This type of noise can lead to inconsistent signal generation or random charging during loading/de-loading cycles. Finally, the contact noise originates from a variable contact area, pressure, or surface property under cyclic mechanical deformations. These composites have various applications, such as health monitoring, wearable electronics, and robotics [39]. Therefore, adding graphene to strain sensors paves the way for innovative applications in various fields, including structural health monitoring.
[iii] Carbon nanotubes: Carbon nanotubes with cylindrical morphology are also known as robust fillers used in reinforcing rubber matrix. They can be single-wall (SWCNT) or multi-wall carbon nanotubes (MWCNT). When added to a rubber matrix, the carbon nanotubes offer a great combination of mechanical, electrical, and thermal properties [40]. Therefore, the carbon nanotubes enhance the performance and sensitivity of strain sensors, enabling their use in various advanced applications. Moreover, these carbon nanotubes exhibit a high aspect ratio, i.e., high length-to-diameter ratio. This high value helps enhance interaction with the polymer matrix and improves load transfer [41]. The role of carbon nanotubes used in strain sensing involves improved sensitivity. This is based on short response time, high durability, signal stability and high flexibility. These factors can be understood as the networks of carbon nanotubes in rubber matrix altering under mechanical strain, altering the resistance and improving the detection of even small deformations [42]. The carbon nanotubes improve mechanical properties and ensure that sensors can withstand repeated bending and stretching cycles that are crucial for robust wearable applications.

Finally, the carbon nanotube networks assist in achieving stable electrical signals, thereby reducing noise and improving output signals more accurately [43]. These carbon nanotubes added in the rubber matrix are useful for various applications. These are health monitoring, wearable electronics, and medical uses such as implants. Overall, the ability of carbon nanotubes assists in improving sensitivity, durability, and signal stability [44]. Moreover, these composites help in enabling the development of flexible and miniaturized sensors. These features make them a vital component in advancing strain sensor technology.

[iv] Carbon nanofibers: Carbon nanofibers are also cylindrical in morphology like carbon nanotubes, with a diameter ranging from 10–100 nm and lengths up to several micrometers. Therefore, they have a large aspect ratio with the combined properties of carbon nanotubes and traditional carbon fibers [45]. Their properties include high tensile strength, thereby contributing strong mechanical reinforcement to the rubber matrix. Moreover, they offer an optimum balance to strength, flexibility, and stretchability. These are critical for strain-sensing measurements [46]. Carbon nanofibers also offer high electrical conductivity, thereby contributing a strong piezoresistive effect that is crucial for strain sensing. Thus, the change in resistance is critical under the change in mechanical strain, enabling high sensitivity for strain sensors [47]. Moreover, the high thermal conductivity of carbon nanofibers makes them ideal for heat dissipation in rubber composite under mechanical strain [48]. Therefore, carbon nanofibers make the composites thermally stable under large mechanical deformations. As discussed already, carbon fibers have a great impact on strain sensitivity when used in sensing applications. Some other crucial prospects are enhanced piezoresistive sensitivity, high durability, and electrical signal stability [49]. Therefore, they are important for applications such as structural health monitoring, wearable electronics, and robotics.
[v] Fullerenes: Fullerenes are a well-known form of carbon in which the carbon atoms are arranged in spherical, ellipsoidal, or tubular structure. The most commonly studied fullerene is C60, a molecule that is composed of 60 carbon atoms arranged in a hollow sphere [50]. Fullerenes offer properties that make them useful for various advanced applications, such as strain sensors. For example, a fullerene with a robust structure helps composites achieve high mechanical strength due to their stable and symmetrical structure [51]. Like other carbon forms, fullerenes offer a good piezoresistive effect, making them suitable for sensing applications. In other cases, fullerenes can be chemically modified to improve their compatibility with different polymer matrices. This process helps in improving their dispersion and interaction within the composite material [52]. From a sensing perspective, fullerenes offer large strain detection that occurs from resistance change under mechanical strain. Moreover, they offer good mechanical reinforcement, flexibility, mechanical stability against fatigue, and noise reduction. The noise reduction and consistent signals help in obtaining error-free and more accurate data [53]. Therefore, their ability to improve sensitivity, durability, and signal stability. Also, they enable the development of flexible and miniaturized sensors, making them a valuable component in advancing strain sensor technology.

The addition of these materials makes the electrode electrically conductive and can offer a quick response to external stimuli. Moreover, these composite materials need to be biocompatible, non-toxic, and lightweight. They also need to have high fatigue resistance to be useful for sensing prototypes [54]. Thus, combining these robust composite materials during fabrication helps in achieving diverse and innovative physical activity monitoring sensors for in demand applications [55,56]. Therefore, this review paper describes the prospects of the recent literature on physical activity monitoring sensors. This review study reports the sensing of various physical activities like sports activities of human beings, including boxing, tennis playing, running, squatting, etc. [57,58]. It also summarizes the prospects of sports sensors on finger pressing or finger bending, etc.

The first section includes a brief introduction about these sensors and the material used during their fabrication. Finally, the advantages and challenges of these sensors are discussed in view of commercialization. Then, the next step was to describe the fabrication procedure of such sensors from assembly to full real-time monitoring. After discussing their fabrication, some critical mechanical and electrical properties were discussed. These mechanical properties are useful for long-term durability and resistance to mechanical failure or fatigue under continuous mechanical deformation. However, the electrical properties report the understanding of the resistance change, response time, and gauge factor in physical activity monitoring sensors. Then, the final part of the review is focused on the industrial applications of these sensors. This part focuses on the different functionalities of various types of sensors for monitoring various types of physical activities. The studied sensors include a review study on physical monitoring sensors for various uses. For example, we report the sensing of sports activities like boxing or physical activities like walking, squatting, or running. The other parts of this review include important investigations on energy generation for self-powered devices under different mechanical loads. Finally, the main aspect of fracture toughness for higher durability in sensors was reviewed. The study shows that the use of materials with fracture toughness is ideal for the use of composites for fabricating sensors with high durability.

### Critical Overview of Rubber Composite-Based Sensors

The rubber composite-based sensors have emerged as a vital class of flexible, stretchable, and wearable sensing materials. These sensors are gaining increasing attention in the fields of soft robotics, healthcare monitoring, and human–machine interfaces. These sensors leverage the mechanical resilience and elasticity of rubber matrices. These matrices are combined with the electrical conductivity of embedded fillers such as carbon black, graphene, carbon nanotubes, or metal nanoparticles. The synergy between mechanical and electrical properties enables rubber composites to exhibit external stimuli. These are mechanical strain, external pressure, or temperature and result in measurable electrical signals. Despite their promising features, the development of rubber composite-based sensors faces several challenges. Among these challenges, the filler dispersion, long-term stability, sensitivity, and hysteresis are in focus. Moreover, repeatability remains critical in determining sensor performance, and the scalability and cost-effectiveness of fabrication techniques continue to impact their commercial viability.

This review aims to evaluate the current advancements critically about the key material systems and fabrication strategies. Moreover, the present review discusses the sensing mechanisms and application domains of rubber composite-based sensors. It also addresses the limitations and open research questions, providing insights into potential pathways for future development in this rapidly evolving field. These sensors have found applications in wearable health monitoring, such as monitoring sports injuries. The other applications include heart-beat monitoring, soft robotics, prosthetics, and electronic skins. Moreover, with the advancements in nanotechnology, 3D printing and self-healing materials are expected to improve the performance of these sensors. There is also a growing interest in multifunctional composites that can sense multiple parameters simultaneously. These parameters are pressure, temperature, and systems with wireless or self-powered functionalities. Overall, these rubber composite-based sensors are at the forefront of flexible and wearable electronics. This is due to their unique combination of mechanical flexibility and tunable electrical properties. However, addressing issues of long-term stability and environmental robustness is essential. Fixing these issues through self-healing and choosing the right material during fabrication is important. Finally, their transition from laboratory prototypes to real-world applications will need to overcome challenges. These challenges are filler dispersion, filler rubber compatibility, and the great interface between filler and rubber matrix.

## 2. Filler Characterizations

### 2.1. Structural Features of the Carbon Nanomaterials

**Figure 1:**
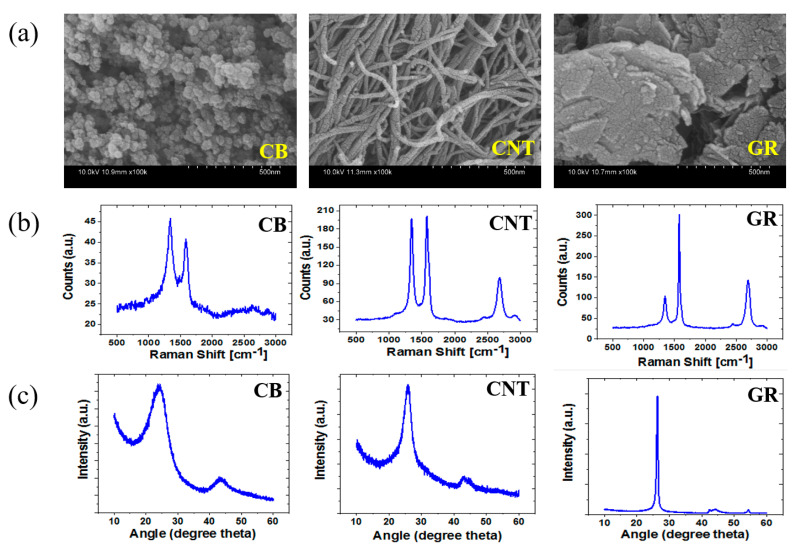
(**a**) SEM of carbon nanomaterials such as CB, CNT, and GR. Here, CB shows oval-shaped morphology, CNT shows tube-like morphology, and GR shows sheet-like morphology; (**b**) Raman spectra of carbon nanomaterials, showing a D-band at 1350 cm^−1^, G-band at 1580 cm^−1^, and 2D band at 2700 cm^−1^. However, the 2D band is absent for CB and the 2D band in GR changes with a change in the number of graphene layers stacked; (**c**) XRD of carbon nanomaterials showing a broad peak around 2θ of 24° to 26° corresponds to the (002) plane of graphite. Reproduced with permission from Elsevier [64].

The morphology of the carbon nanomaterials, namely carbon black, carbon nanotubes, and graphene, plays a crucial role in influencing the properties of the composites. Therefore, understanding their morphological features is important to obtain properties of interest [59]. Starting with carbon black, Figure 1a shows that carbon black particles consist of quasi-spherical primary particles with diameters typically ranging from 10 to 500 nm. These primary particles fuse together to form larger aggregates, which are the basic morphological units of carbon black [60]. These aggregates can further cluster into loosely bound agglomerates, which can be broken down during processing. The carbon black particles can be useful as a source of reinforcement and electrically conductive additives in rubber composites [61].

Figure 1a shows the morphological features of carbon nanotubes. As described already, the carbon nanotubes are cylindrical tubes composed of rolled-up sheets of graphene. These carbon nanotubes can be single-walled or multi-walled, depending upon the number of layers rolled into a tube [62]. Typically, single-walled CNT has an approximate diameter of 1 nm, while multi-walled CNT have diameters from a few nm up to 10 nm. In addition, carbon nanotubes exhibit a high length-to-diameter ratio, which is the aspect ratio, sometimes exceeding 1000 [63]. This high aspect ratio contributes to the exceptional mechanical and electrical properties of composites. Carbon nanotubes also have various commercial uses such as reinforcing agents, electronic devices, energy storage gadgets like supercapacitors, and strain sensing [64].

Similarly, Figure 1a shows the morphological features of graphene. As already stated, graphene is a two-dimensional single sheet of carbon atoms that are arranged in hexagonal lattices. Graphene has an extremely high surface area, and this feature makes it useful as a source of reinforcement in rubber matrices [65]. In some cases, the functionalization of graphene sheets is performed to make it compatible with the rubber matrix, and improved interfacial interactions are expected. The improved interface makes the composite robust, and high reinforcing properties are expected [66]. Therefore, graphene can be useful in flexible electronics, sensors, and energy storage applications. Overall, the unique morphologies of these carbon nanomaterials are fundamental to their diverse use. For example, they are useful in improving properties for a wide range of applications [67]. Also, carbon black’s aggregated structure, carbon nanotubes’ high aspect ratio, and tubular form contribute to their respective roles in enhancing materials and devices. Moreover, graphene’s two-dimensional planar structure assists in achieving high properties for the application of interest.

Raman spectra are well known as a powerful tool in studying carbon nanomaterials. The Raman spectra assist in providing insights into structural and electronic properties [68]. These properties are described by analyzing the vibrational modes of the carbon atoms. Raman spectra in Figure 1b show that the Raman spectra of carbon black exhibit a D-band at 1350 cm^−1^ [64]. This band originated from breathing modes of sp^2^ carbon atoms in rings and indicates defects and disorder in the structure of carbon black. The intensity of the D-band increases with the amount of disorder and defects in the structure of carbon black [69]. Another characteristic G-band located at 1580 cm^−1^ corresponds to the in-plane vibration of sp^2^ carbon atoms in the graphitic lattice. The intensity of the G-band provides the degree of graphitization; a higher intensity suggests more graphitic or crystalline regions [70]. Similarly, Figure 1b shows the Raman spectra of carbon nanotubes. The D-band is positioned at 1350 cm^−1^, like other carbon materials. It indicates the presence of defects, disorder, and amorphous carbon in the carbon nanotubes [71]. Similarly, the G-band position is at 1580 cm^−1^ and provides information about the electronic structure and the degree of graphitization. Moreover, there is another characteristic 2D band at 2700 cm^−1^. It signifies sensitivity to the electronic properties and can be used to distinguish between single-walled and multi-walled CNTs [72]. Similarly, Raman spectra of graphene were reported in Figure 1b. The results show that D-band and G-band are like other carbon materials. Moreover, the 2D band was also noticed at 2700 cm^−1^. It is a characteristic peak for the number of graphene layers stacked in the graphene flake [73]. For example, a sharp 2D band is characteristic of single-layer graphene.

XRD is well known as a powerful tool that provides the crystallography structure and physical properties of a material. Therefore, it is frequently used to characterize the crystal structure of carbon nanomaterials. Figure 1c provides the XRD of carbon black, and the pattern shows that it is amorphous [64]. It can be stated that the XRD pattern is broad and presents featureless peaks. This broad peak around 2θ of 24° corresponds to the (002) plane of graphitic materials. This feature indicates a short-range order but without a long-range crystalline order [74]. Similarly, the XRD of carbon nanotubes exhibits a prominent peak around 2θ of 26°, corresponding to the (002) plane of graphite. Carbon nanotubes can be briefly useful in electronics, reinforcing agents, and sensors. Finally, the XRD of graphene shows a noticeable peak around 2θ of 26° that relates to the (002) plane of graphite [75]. However, the difference in peak width and intensity is related to the number of graphene layers stacked in the crystalline domain.

### 2.2. Structural Characterizations

**Figure 2:**
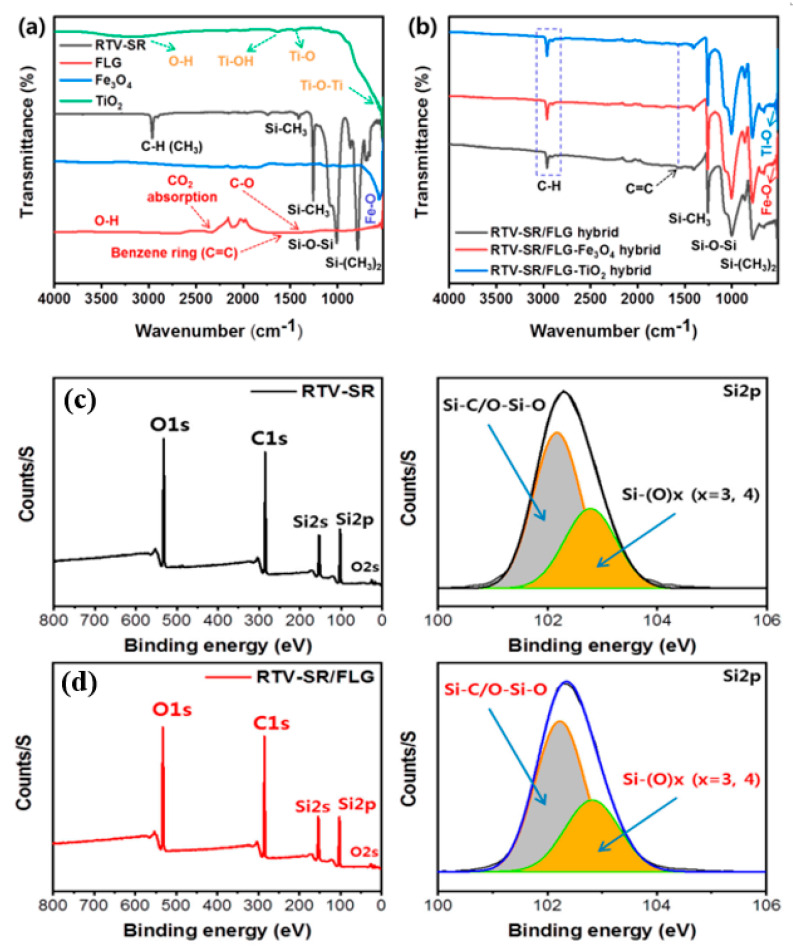
(**a**,**b**) FTIR of carbon nanomaterials and their composites. FTIR of silicone rubber shows functional groups like C-H, Si-CH_3_, Si-O-Si; FTIR of FLG shows functional groups like O-H, C=C, C-O and adsorption of CO_2_; FTIR of iron oxide shows functionalities like Fe-O; FTIR of TiO_2_ shows functional groups like O-H, Ti-OH, Ti-O, and Ti-O-Ti; finally, FTIR of composite sample shows functionalities like C-H, C=C, Si-CH_3_, Si-O-Si; (**c**,**d**) XPS of unfilled and graphene-filled silicone rubber compote samples. Results reveal that silicone rubber contains Si-O-Si and Si-C bonds, indicating silicone polymer networks. Deconvolution results show interactions between graphene and silicone rubber. These interactions can be physical adsorptions or chemical bonding, such as Si-O-Si linkages Reproduced from open access article [76].

FTIR is regarded as a powerful tool for obtaining the molecular composition and structure of the materials. FTIR is used for studying carbon nanomaterials like graphene or silicone rubber, as studied in the present review. Figure 2a shows the FTIR spectra of graphene [76]. The results show C=C stretching vibrations around 1580 cm^−1^, justifying the presence of a graphitic structure. It is known to have originated from the stretching vibrations of sp^2^ hybridized carbon atoms in the graphene lattice [77]. Further, the O-H stretching was located around 3500 cm^−1^ and proposed from functionalization with hydroxyl groups. It can enhance the hydrophobicity and result in higher surface activity of graphene.

C=C stretching is located around 1700 cm^−1^ and indicates the oxidation of graphene [78]. It is proposed to originate from the synthesis method or functionalization process. Similarly, Figure 2b reveals the FTIR of silicone rubber and their composites with respective characteristic absorption bands. They are proposed to originate from the functional groups in silicone rubber. The spectra reveal Si-O-Si stretching vibrations around 1100 cm^−1^. They are originated from stretching vibrations of the siloxane backbone [79]. Another Si-CH_3_ stretching vibration at around 1270 cm^−1^ is known to have originated from the methyl group attached to silicon in the rubber. The C-H band at around 2970 cm^−1^ was proposed to originate from C-H bonds in methyl groups. Thus, FTIR is proposed to be useful for analyzing and validating the chemical composition and structure of both graphene and silicone rubber [80].

XPS is a powerful tool for investigating the surface chemistry of the materials. Analyzing carbon nanomaterials like graphene in silicone rubber composites provides elemental composition. XPS also provide chemical states, interactions, and knowledge about additives [81]. Figure 2c,d show the XPS results of silicone rubber and graphene-filled silicone rubber composites. The results show that the elemental compositions identified are carbon, silicone, and oxygen for both samples. The XPS results further show the presence of functional groups like hydroxyl and carbonyl groups on the surface of graphene [82]. These interactions can be physical adsorptions or chemical bonding, such as Si-O-Si linkages [83]. The results further validate the composite processing’s absence of contaminants or residual solvents.

## 3. Properties

### 3.1. Mechanical Properties

Understanding the mechanical properties of physical activity monitoring sensors is very important. The composites based on elastomers and electrically conducting fillers are often the best candidates [84]. They exhibit good mechanical properties suitable for fabricating physical activity monitoring sensors. These composites are stretchable and flexible and exhibit high sensitivity and reliability [85]. Moreover, they have robust durability in various industrial applications like physical activity monitoring sensors. The other useful mechanical parameters are elasticity, modulus, fracture strain, and hysteresis losses. [86,87]. Moreover, a balance of these mechanical properties is critical to obtaining robust performance for physical activity monitoring sensors. For example, these monitoring sensors exhibit good stretchability and can help in accurately detecting and measuring small changes [88]. They also exhibit an optimum compressive modulus, which helps in exhibiting greater sensitivity to small changes in strain. Their higher tensile strength is critical for enabling the reliability and durability of strain sensors under various loading conditions [89]. Moreover, their lower hysteresis losses as heat dissipation losses help achieve accurate and repeatable measurements. Finally, the composites with high fatigue resistance ensure long-term reliability and durability [90]. Therefore, mechanical properties are critical for physical activity monitoring sensors.

**Figure 3:**
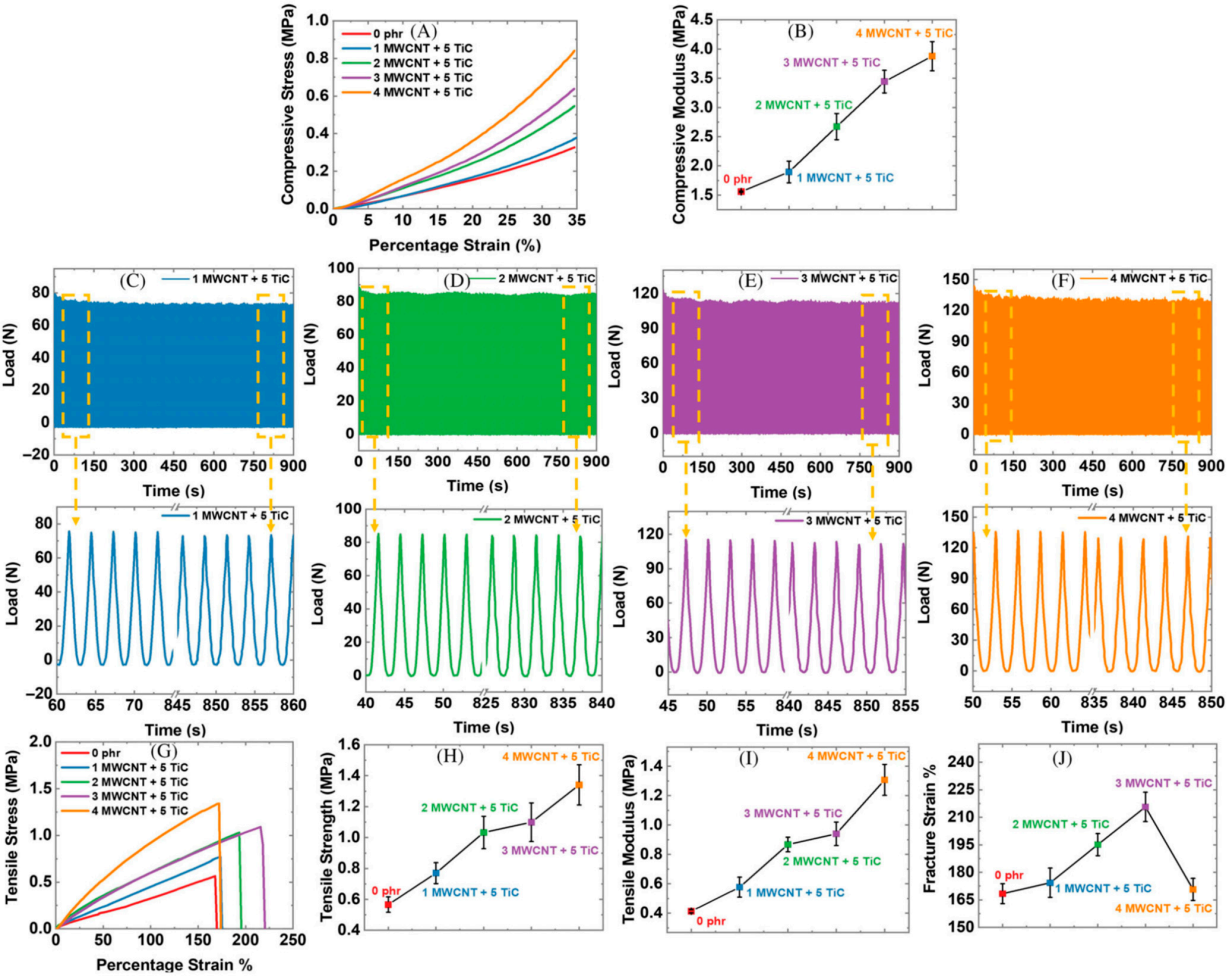
(**A**) Stress–strain profiles under compressive strain. Results show that compressive stress increases with increasing MWCNT + TiC content. Moreover, stress increases almost linearly up to 10%, then increases exponentially until a compressive strain limit of 35%. (**B**) Compressive modulus of different composites. Results show that the compressive modulus increases with increasing content of MWCNT + TiC. (**C**–**F**) Different samples under cyclic compressive loads. Output mechanical load was stable with negligible losses from the initial to final cycles. (**G**) Stress–strain under tensile strain. Stress was higher at higher strain, reaching a maximum before fracture. (**H**–**J**) Tensile strength, tensile modulus, and fracture strain of different composites. These mechanical properties increase with increasing MWCNT + TiC content, reaching the maximum at 4 phr of MWCNT + TiC. However, fracture strain decreases after 3 phr of MWCNT. Reproduced with permission from reference [91].

Figure 3A presents stress–strain curves for different composites to be useful for sensors [91]. The results show two types of behavior. One is an increase in stress with increasing compressive strain. Another is an increasing stress at all percentage strains with increasing MWCNT content in the composite. Firstly, the higher stress resulting from higher strain could be because of higher packing fractions of the ingredients of the composite at higher compressive strain [92,93]. Moreover, the stress increases almost linearly up to 10%, then increases exponentially until a compressive strain limit of 35%. The exponential increase after 10% is due to optimum strain, after which there are higher packing fraction effects. In addition, the higher stress by increasing MWCNT content could be due to higher inter-interfacial interactions [94]. These interactions resist the composites under external mechanical force under the loading phase of strain. During the unloading phase, the interactions re-establish and achieve equilibrium during cyclic mechanical deformations [95].

Similarly, the compressive modulus in Figure 3B increases with increasing MWCNT content, reaching a maximum at 4 phr MWCNT + 5 phr TiC sample. This can be influenced by a higher “reinforcing effect” of MWCNT in the composite. Moreover, the higher modulus is due to efficient and robust interfacial interactions in the composites [96]. Finally, the high properties are due to optimum fabrication methodology, dispersion, orientation, and high aspect ratio of MWCNT [97]. These factors strongly influence the improved compressive modulus with increasing MWCNT content in composites. These aspects are important for achieving high performance for physical activity monitoring sensors for mechanical stability and durability [98,99]. Among the various main mechanical factors, mechanical stability is critical for physical activity monitoring sensors. Thus, Figure 3C–F shows the mechanical stability under compressive cycling for different composites. The results reported by Manikkavel et al. [91] show that under continuous compressive cyclic strain, the output mechanical load was stable with negligible losses from the initial to final cycles. This behavior supports their use of physical activity monitoring sensors [100]. These sensors require higher mechanical stability and critical fatigue resistance against mechanical failure. This can be correlated to better load transfer, thereby making the composite more resistant to mechanical deformation [101]. Moreover, the desired modulus and sensitivity can obtain better performance for physical activity monitoring sensors.

Figure 3G reports stress–strain curves under tensile strain. Like previous results in Figure 3A, the stress was higher at higher strain, reaching a maximum before fracture. This could be a result of the breakdown of interfacial interactions in in-rubber structures. These features are also due to other interactions within the composites under increasing tensile strain [102]. Other parameters, like tensile modulus, tensile strength, and fracture strain, can be derived from these stress–strain curves and reported in Figure 3H–J. Most of the results agree well with the stress strain in which the modulus and tensile strength increase while fracture strain falls after 3 phr MWCNT 5 phr TiC. The improved mechanical properties with filler addition are well known, and there are several reasons for such behavior. For example, the higher “reinforcing effect” of MWCNT in composite helps in achieving higher mechanical properties [103]. Higher interfacial interactions with the addition of MWCNT in the composites assist in achieving higher mechanical properties. Finally, good fabrication technology, filler dispersion, and the high aspect ratio of MWCNT in composite help in achieving higher mechanical properties [104].

Finally, the fall in fracture strain after 3 phr MWCNT + 5 phr TiC in Figure 3J could be due to the partial aggregation of filler particles in composite materials. It is essential to understand the modulus of the composites for their usefulness as physical activity monitoring sensors [105]. Moreover, the tensile strength is crucial for composite materials. It provides important information about the quantity of stress a composite can withstand before mechanical failure or fatigue [106]. Moreover, the fatigue behavior is important for understanding the reliability and durability of sensing devices. Finally, the fracture strain is useful to understand the ultimate elongation the composite can withstand before failure. This is one of the most important aspects of the use of composites for various physical activity sensing applications [107].

### 3.2. Electrical Properties

The physical activity monitoring sensors are generally based on elastomers that are lightweight and stretchable under external mechanical deformations [108]. These physical activity monitoring sensors have key factors like resistance change, response time, and gauge factors while evaluating their performance [109]. These factors are crucial for understanding the performance of a strain-sensing device. The strain sensors work on the principle of piezoresistance in which the resistance change happens in response to applied strain [110]. Such a resistance change is usually proportional to the applied strain and depends upon the material’s piezoresistive coefficient. Then, the response time refers to the ability of the strain sensor device to respond to external stimuli [111]. Generally, a faster response time is required for dynamic applications. Therefore, the response time is related to mechanical properties, sensor design, and the electronics of the strain sensor. Finally, the gauge factors measure the sensitivity and quantify the resistance change against the strain [112]. Technically, it is defined as the ratio of relative change in resistance (ΔR/R) to the applied strain (Δε). Thus, these above factors are useful in understanding the performance of strain sensors. For example, using materials with higher piezoresistive coefficients can enhance the resistance change per unit strain, thereby influencing the sensitivity. Overall, researchers need to develop a strain sensor with optimum performance, such as desirable resistance change, faster response time, and optimum gauge factor [113].

**Figure 4:**
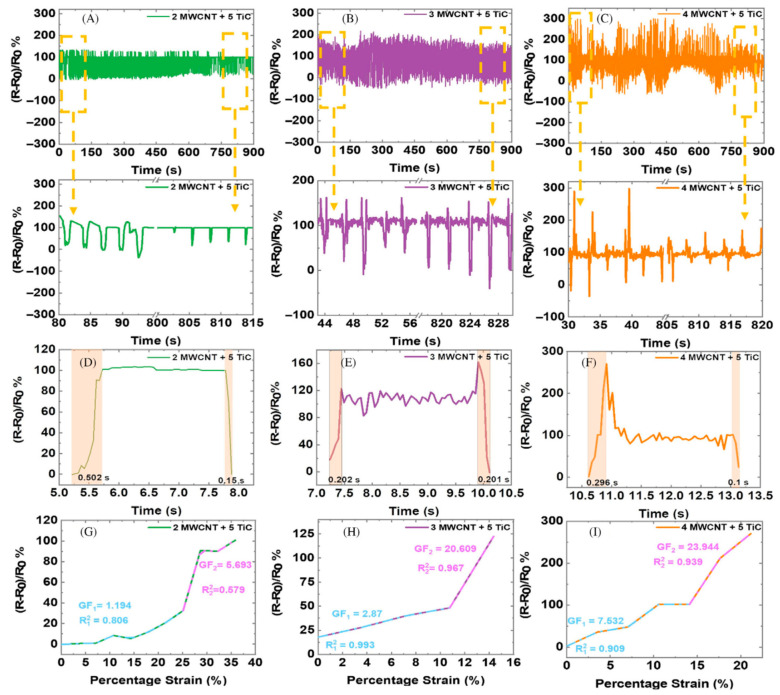
(**A**–**C**) Relative resistance change for different samples. Results show a higher change in resistance by increasing MWCNT + TiC in composites. Effective piezoresistive exhibit can be understood by composites based on SR matrix and MWCNT and TiC. (**D**–**F**) Response time for different composites. Response time decreases with increasing MWCNT content. For example, response time of loading phase at 2 phr MWCNT + 5 phr TiC was around 0.502 s, and it decreased to 0.202 s at 3 phr MWCNT + 5 phr TiC. (**G**–**I**) Gauge factor of different samples. Gauge factor was 1.19 at 25% strain and increased to 5.6 by increasing compressive strain to 35%. Reproduced with permission from reference [91].

Considering these aspects, Park et al. [91] fabricated composites by adding different propositions of MWCNT and fixed 5 phr of TiC into silicone rubber. Finally, these composites were studied for their use in strain sensing by reporting resistance change, response time, and gauge factors [114]. Figure 4A–C report the resistance change by increasing the MWCNT content in the SR matrix. The piezoresistive effect can be understood by composites based on SR matrix and MWCNT and TiC. This helps in achieving higher change in electrical resistance in response to mechanical strain [115]. This can be understood in more detail as the formation of electrically conductive pathways in composite by MWCNT and TiC into SR matrix. Thus, as the composite undergoes compressive mechanical deformation, there is a change in resistance [116]. Finally, it results in enhancing the piezoresistive effect and amplifying the change in resistance for a given level of strain. Another mechanism for achieving higher piezoresistivity is related to filler percolation phenomena [117]. For example, increasing the MWCNT content beyond the threshold helps the composite material transition to a more conductive state. Finally, this assists in making the composite more sensitive to changes in strain-induced resistance [118]. Moreover, the with change in resistance, the sensitivity of the composite can be adjusted by changing the content of MWCNT in the SR matrix. This can be adjusted by tuning MWCNT content for desirable performance. This tuning allows for optimization factors like sensitivity, linearity, and mechanical properties of the final composite [119,120]. Overall, the mechanical stability is important in affecting the fatigue and durability properties of these composites. For example, the mechanical stability of these composites helps to withstand mechanical strain without failure, ensuring reliable performance as a strain sensor over extended usage [121].

In addition to change in resistance, the response time is of utmost importance to study strain sensors. Figure 4D–F present the influence of increasing MWCNT content on the response time of the composites. The results show that the response time decreases with increasing MWCNT content. This behavior is proposed because of the higher electrical conductivity of the composites with an increase in MWCNT content [122]. The transmission of electrical signals or the sensing of changes in electrical properties results in a smaller response time. This can be understood more efficiently as the higher electrical conductivity helps in faster transmission of electrical signals [123]. For example, the resulting electrical response under mechanical deformations can be detected more quickly in materials with higher conductivity. Moreover, the sensing performance is improved with composite with higher electrical conductivity [124]. For example, the time taken to detect changes in resistance due to mechanical deformation can be reduced in more conductive materials. In many cases, the higher electrical conductivity helps in reducing electrical noise or interference in composite materials [125]. It helps to ensure faster and more reliable signal processing and data transmission. It is useful where accurate and timely detection of signals is critical, such as in medical devices [126].

Finally, the gauge factor and linearity of the composites are understood by the results reported in Figure 4G–I. It is well known that the gauge factor depends critically on the magnitude of strain and electrical conductivity of the composite studied. This is expected due to an increase in the packing fraction of the additives in the composite during fabrications. The higher packing fraction influences the resistance change, and thereby the higher gauge factor was witnessed [127,128]. In another case, with increasing MWCNT content, the gauge factor and linearity are also better. It is also due to higher electrical conductivity by increasing MWCNT content in the composite. The higher electrical conductivity improves the charge carriers within the composite and allows more efficient transmission of electrical signals [129].

Thus, the process finally influences the change in resistance, thereby leading to better linearity and higher gauge factors. Moreover, higher electrical conductivity helps in enhancing sensitivity to external stimuli. Finally, it enhances the accuracy and reliability of strain-sensing applications [130]. In another case, these strain sensors should exhibit a linear response, where the resistance change is directly proportional to the applied strain over a wide range of strain values. Here, the higher electrical conductivity can contribute to improved linearity by minimizing non-linear effects such as mechanical hysteresis losses [131]. For example, Park et al. [91] show that the linearity was 0.81 for 1 phr MWCNT with 5 phr TiC, and it increases to 0.91 for 3 phr MWCNT with 5 phr TiC. Therefore, electrical conductivity plays a crucial role in enhancing the gauge factor and linearity of composites used in strain-sensing applications.

## 4. Applications

### 4.1. Physical Activity Monitoring Sensors Under Tensile and Compressive Strain

In composite materials that are used in strain sensing, the change in resistance can vary based on composition, structure, and electrically conductive properties [132]. For example, under compression strain, the distance between conductive pathways of filler, such as MWCNT, changes in the composite. This leads to a change in resistance and, thus, different sensing performance [133,134]. This case is similar to graphene-based composites, in which the electrically conductive pathways became denser, resulting in resistance change and thus better-sensing properties. Similarly, the change in resistance for CNT and graphene-based composites is higher, thereby resulting in resistance change and lower sensing capacity [135]. Therefore, specific resistance change is based on the type of strain primarily driven by changes in the conductive pathways within the material. Park et al. [136] report a strain sensor fabrication by solution mixing of silicone rubber with carbon nanotube. This mixing was performed for 10 min following the addition of the vulcanizing agent and kept at room temperature for 24 h for vulcanization. After vulcanization, the final composites were tested for properties under tensile and compressive strain.

**Figure 5:**
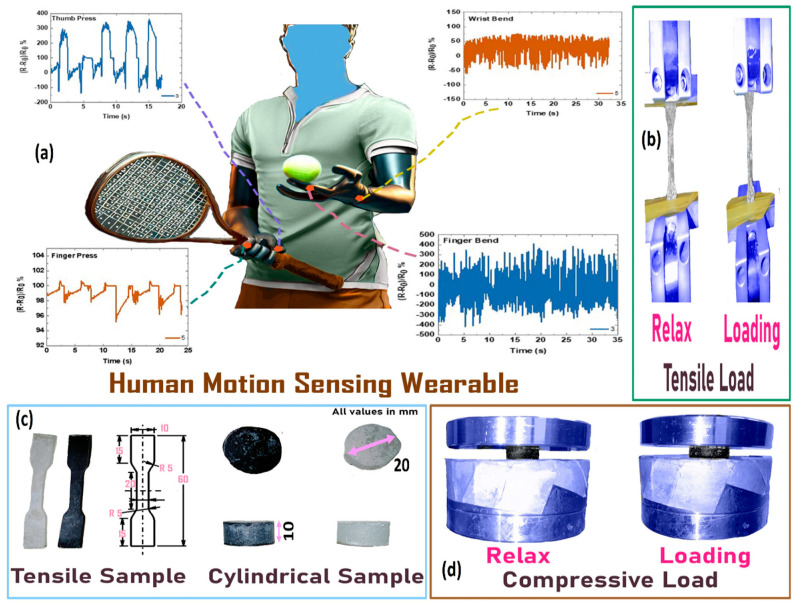
(**a**) Relative resistance change for various human motion sensing. Change in resistance was higher during thumb pressing, mimicking compression strain on cylindrical sample. However, it was highest for finger bending that mimics tensile strain. (**b**) Sample subjected to tensile load. These researchers performed strain sensing using cylindrical samples for compressive tests and dumbbell samples for tensile testing. (**c**) Details of sample geometry. Cylindrical samples were 10 mm thick and 20 mm wide. Tensile test’s dumbbell sample has a thickness of 2 mm and a gauge length of 20 mm. (**d**) Sample subjected to compressive load. Relaxation and loading mechanisms are reported. Reproduced with permission from Wiley [136].

Figure 5a shows the outline of the wearable sensor in the human body. The real-time sensing responses can be performed under thumb pressing, finger pressing, finger bending, and wrist bending [137]. For strain sensors utilizing composite materials, those used in wearable devices can induce varying responses in terms of resistance change [138,139]. Figure 5b shows the relaxation and loading under tensile strain from a universal testing machine. These strain types can be understood more extensively as thumb or finger pressing states, as when pressure is applied with the thumb, it results in localized compression on the sensor surface. This compression can result in a decrease in resistance change, thereby increasing conductive pathways in composite elements [140]. Then, the finger bending or wrist bending results in inducing complex strain on the sensor surface. The dimensions of cylindrical and dumbbell samples are presented in Figure 5c. Finally, Figure 5d shows relaxation and loading under compressive strain. Generally, the bending process results in variable compressive or tensile strain across the sensor, resulting in varied response in resistance.

### 4.2. Sensing of Physical Activity Like Boxing

#### 4.2.1. Overview of Deep Learning and Data Analytics for These Sensors

This review introduces the use of deep learning for use in physical activity monitoring sensors. Here, the integration of deep learning and data analytics with composite-based sensors has opened new frontiers in intelligent sensing systems. These sensors use flexible and use multifunctional materials such as rubber composites and electrically conducting fillers. They generate rich, complex data streams in response to various physical stimuli, including pressure, strain, temperature, and motion. Moreover, exploitation of the data from physical activity like walking, running, etc. is performed for accurate sensing. The other functionalities involve the recognition, decision-making, and advanced data-driven approaches. Here, deep learning, a subfield of machine learning based on artificial neural networks, can be explored. It has proven especially powerful in handling large, high-dimensional datasets. It can automatically extract hierarchical features from raw sensor signals. Then, it enables robust classification, regression, and anomaly detection without manual feature engineering. This is particularly valuable for wearable and soft sensors, where signal patterns may be non-linear, noisy, and highly dynamic. Finally, complementing this, data analytics techniques are used. These are statistical modeling, signal processing, and real-time data fusion. They enhance sensor functionality by improving accuracy and enabling sensor calibration. Finally, they help in supporting multi-sensor integration. These technologies transform composite-based sensors from passive signal generators into smart systems. For example, deep learning is capable of adaptive learning, real-time feedback, and predictive analytics. This integration is crucial for applications such as human motion analysis. The other applications are health monitoring, soft robotics, and structural health diagnostics.

#### 4.2.2. For Real-Time Monitoring for Boxing Sensor

**Figure 6:**
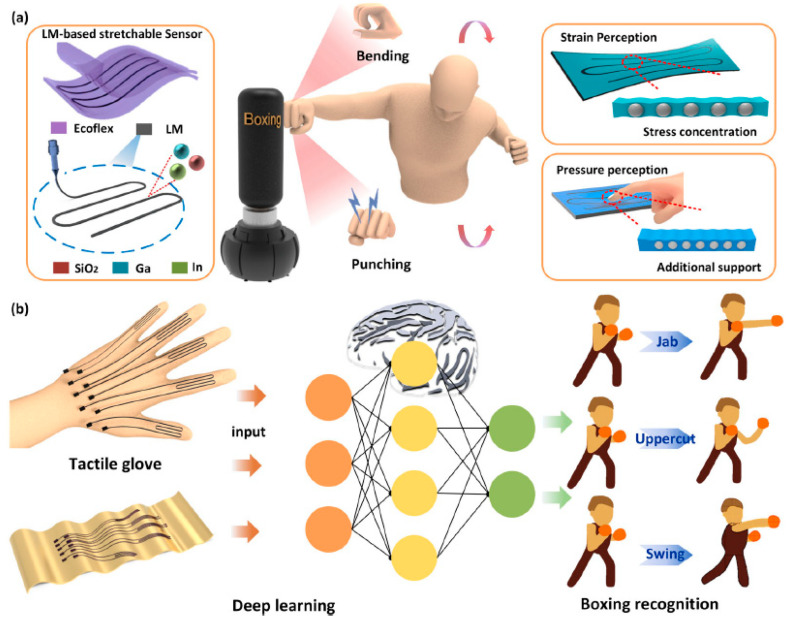
(**a**) Fabrication of sensor and their prospects. Device was developed by 3D printing of liquid metals on silicone rubber with the brand name “Ecoflex”. (**b**) Wearable sensor, deep learning, and real-time monitoring of boxing. With future advancements, deep learning and data analytics will play an increasingly central role in unlocking the full potential of composite-based sensor systems. Reproduced with permission from open access article [149].

The real-time sensing of physical activity involves the integration of smart materials as sensors to monitor physical sports activities such as human motion [141]. They are useful for various industrial applications, such as sports performance analysis and healthcare monitoring [142,143,144]. Thus, integrating smart materials that are active in piezoelectricity [145], piezoresistivity [146], or capacitive elements [147] within the sensor is useful for sports activity monitoring. These sensors are useful for measuring changes in relative resistance under mechancial stimuli and transmit data through connections for real-time analysis [148]. These sensors are wearable in clothing, footwear, and hand gloves as reported in Figure 6a [149]. The author reports the monitoring of human motions or sports activities like boxing for biomechanical analysis. The viscosity, modulus, and tensile strength can be improved by mixing SiO_2_ micro-particles into liquid metals [149]. These aspects allow the sensor used for sports monitoring with high sensitivity with increasing gauge factors. Here, the gauge factors increase from 5.72 at 100% strain to 23.91 at 300% strain. After printing, the sensor was fabricated onto the textile glove for real-time monitoring of the prototype. These sensory arrays are integrated with tactile gloves for evaluating boxing motions like jab, swing, uppercut, etc. [149]. This can be further understood in Figure 6b. Such analysis involves an analysis of sports performance optimization or injury prevention mechanisms. Finally, the real-time data can be obtained from WiFi, Bluetooth, or cloud-based platforms for analysis and visualization. Finally, the signal processing algorithms are employed to understand sensor data. The real-time feedback from physical activity and performance metrics has an accuracy of up to 90.5% [149].

**Figure 7:**
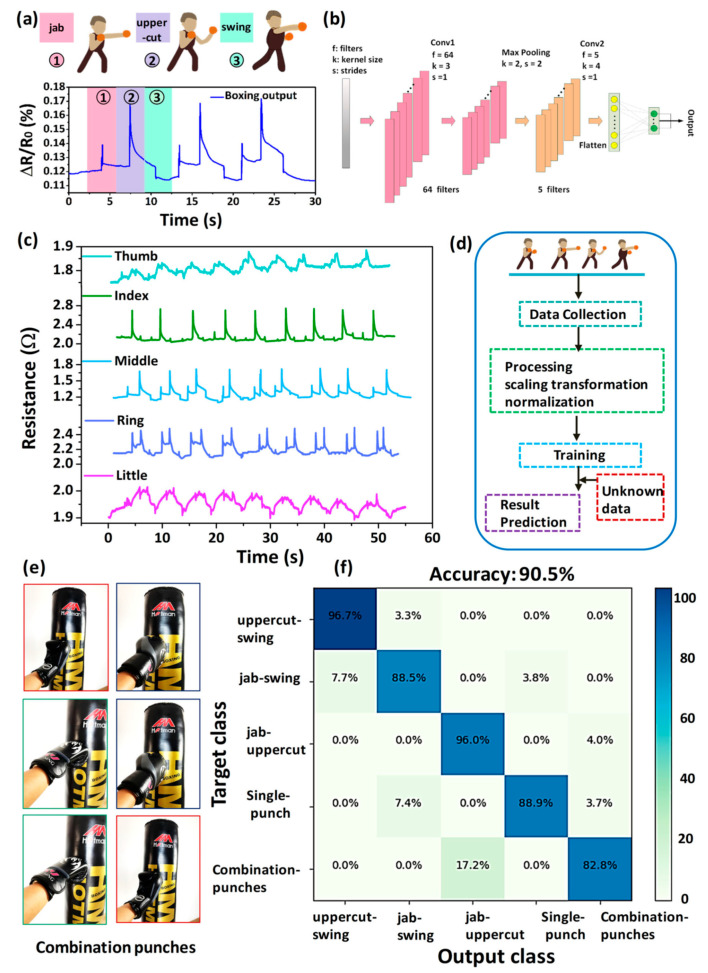
(**a**) Different configurations in boxing. This sensor was fabricated by 3D printing liquid metals on silicone rubber. Mechanical properties like modulus and tensile strength were improved by mixing SiO_2_ and micro-particles into liquid metals as reported. (**b**) Filtering process of data. Whole data sample was used in 150 sets, and 30 sets for each punch were reported. (**c**) Resistance change of different human motions during boxing activity. (**d**) Data collection and result prediction process. (**e**) Target class and accuracy of the system. Reported results indicate that boxing indication shows an accuracy of >90%. Overall, resistance to change during different human motions enables real-time monitoring of physical activities. (**f**) Classification test confusion matrix of boxing recognition derived from the tactile glove. Reproduced with permission from open access article [149].

A physical activity like boxing can be monitored with a physical activity sensor with an accuracy of >90% [149]. These sensors represent a powerful tool for promoting advanced research for sports like monitoring jabs, uppercuts, or swings in boxing. These sensors can harness sensor technology, data analytics, and health monitoring solutions [150]. Moreover, these sensors are equipped to sense various advanced sports systems, mechanical motions, or physiological parameters of human health [151]. Moreover, such sensors can capture data on movements, behaviors, and inertial applications. The inertial sensors include accelerometers, global positioning systems, and health monitoring like changes in heartbeat during sports activity [152,153]. Therefore, Xiao et al. [149] report the monitoring of physical activity like boxing through such sensors. Figure 7a shows the monitoring of change in relative resistance during jab, uppercut, and swing operations during boxing using the composite-based physical activity sensor. The reported real-time monitoring results show that the change in relative resistance was higher for the uppercut, medium for the jab, and lower for the swing. The higher resistance change of the uppercut could be proposed due to the higher magnitude of strain that results from higher mechanical deformation and, thus, higher resistance change [154]. For example, a smart tactile glove can be used to evaluate the recognition of a particular activity through an algorithm, as seen in Figure 7b. The final punch was divided into a training set (90%) and a testing set (10%) by adopting the Convolutional Neural Network (CNN) model. Figure 7c reports the real-time monitoring of resistance change against various types of hand motions like finger bending. These reported results show that the resistance to change was highest for the middle finger and lowest for thumb bending, as suggested by Zhang et al. [155]. The results are consistent and reproducible concerning the type of bending.

It is well known that the resistance changes sensors often rely on piezoresistive mechanisms. For example, under mechanical motions of bending or pressing, the electrical resistance changes proportionally depending upon the magnitude of mechanical stimuli [156]. This change in resistance can be measured and correlated with specific human motions like finger pressing or bending during boxing [149]. The wearable sensors embedded in gloves can detect changes in resistance corresponding to repetitive motions like jabs, uppercuts, or swings in boxing. Figure 7d shows the signal processing steps that include data collection. The collected data can be transmitted through the wireless platform, processing scaling transformation of data, and final validation of the results. Finally, Figure 7e shows the optical image of real-time boxing, and Figure 7f shows the confusion map of the classified results. By capturing and quantifying changes in resistance, these sensors can provide valuable insights into movement patterns and sports activities like boxing. Moreover, the change in resistance is the fundamental principle of motion sensing [157]. It, therefore, allows for the detection, quantification, and interpretation of various sports activities like boxing.

### 4.3. Monitoring Human Motions Like Squatting, Walking, or Running

**Figure 8:**
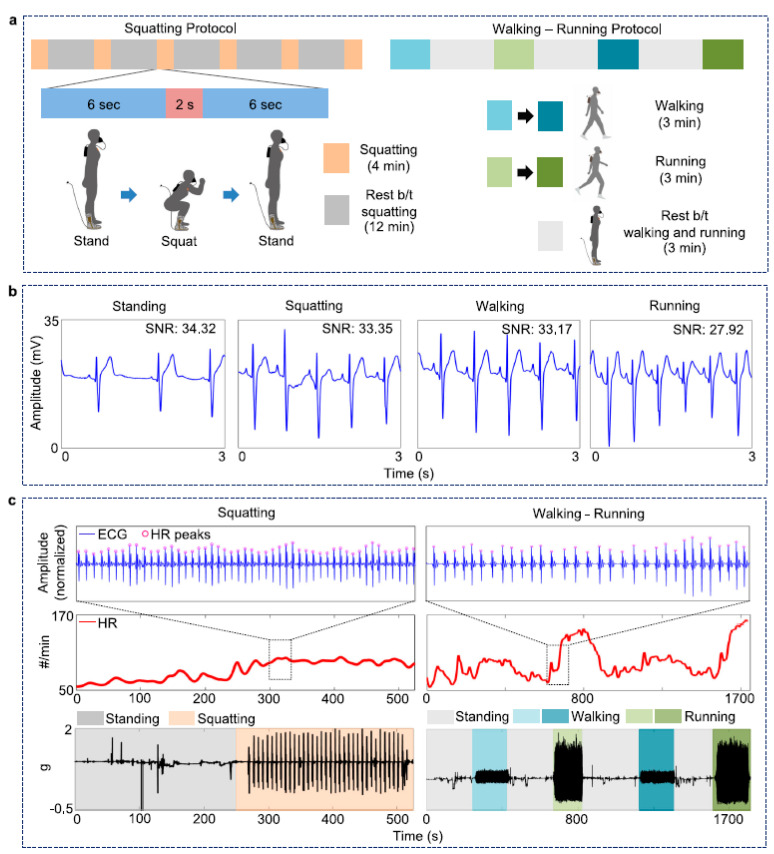
(**a**) Squatting, walking and running protocols. Respective data, like electro-cardio graphs (ECG) obtained through these sensors from physical activities, can be collected by involving algorithms. These algorithms analyze sensor data to recognize and quantify specific movements. (**b**) Output of ECG from different human motions. (**c**) Total protocols of one set of the activities involved. Normalized ECG peaks provide respective respiration rate from protocols. Final results evaluate mechanical stability of sensor during real-time monitoring with reduced noise and remarkable ECG signal quality. Reproduced with permission from open access article [161].

Physical activity can be monitored by enabling the real-time tracking of human motions like squatting, walking, or running. The sensor involved helps in providing insights into biomechanics and performance metrics and is often helpful to avoid possible injury [158]. As discussed already, the sensors used in physical activity monitoring involve various useful technologies. These technologies are like accelerometers, gyroscopes, or in some cases equipped with global positioning systems. These sensors are generally integrated with various wearable devices like smartwatches or fitness trackers [159]. The tracker’s examinations of parameters like joint angles, acceleration, velocity, and force distribution are useful for potential improvements during monitoring [160]. Yeo et al. [161] reported human motion monitoring like squatting, walking, or running. The detailed protocols of the motions are presented in Figure 8a. The figures detail the squatting, walking, and running protocols for a season and respective timings during data collection. These movements are like squatting, walking, or running [162]. The raw ECG quality, as shown in Figure 8b, shows the overall output from the different human motion protocols. The total protocols of one set of the activities involved are reported in Figure 8c. The final results can be monitored by filtering the data to remove noise-related errors and analyzed to target applications like squatting, walking, or running. Moreover, the sensors can provide feedback on movement patterns and ensure they adhere to prescribed exercises [163]. One key advantage of these sensors is their ability to provide real-time feedback to users. This quality helps the user to immediately correct the feedback and helps to adjust and achieve their goals more effectively. Irrespective of the many advantages of such sensors for monitoring human motions, they also have some limitations. For example, accuracy issues, data interpretation complexities, and user comfort concerns. Moreover, ensuring data privacy and security is crucial, especially when dealing with monitoring [164,165].

### 4.4. Self-Powered Energy Harvesting Under Different Mechanical Loads

This self-powered green energy harvesting is promising and hoped for a polluted environment and thus studied extensively. There are various ways of harvesting mechanical energy like vibrations, motion-induced, strain-based, or under different mechanical loads through piezoelectricity [166]. These can be understood more efficiently as when the composites were subjected to external vibration mechanical loads from sources like machinery, vehicles, or natural sources that can generate energy through piezoelectric or electromagnetic mechanisms [167,168]. These piezoelectric materials can be lead zirconate or polyvinylidene fluoride that generate energy in response to external mechanical stimuli or vibration sources. However, electromagnetic composites that contain electromagnetic materials generate energy through the motion-induced variation of magnetic fields within the composite structure [169]. Motion-induced energy harvesting involves the generation of voltage through human or vehicle motions. The energy generation in such cases uses mechanisms like piezoelectric, triboelectric, or electromagnetic induction for energy harvesting [170]. The triboelectric mechanisms involve the use of the contact and separation of dissimilar materials within the composite structure. This prototype finally assists in generating electrical charges through the triboelectric effect [171]. The electromagnetic induction mechanisms involve energy generation when composite material is subjected to different magnetic fields due to relative motions, as described by Lai et al. [172].

**Figure 9:**
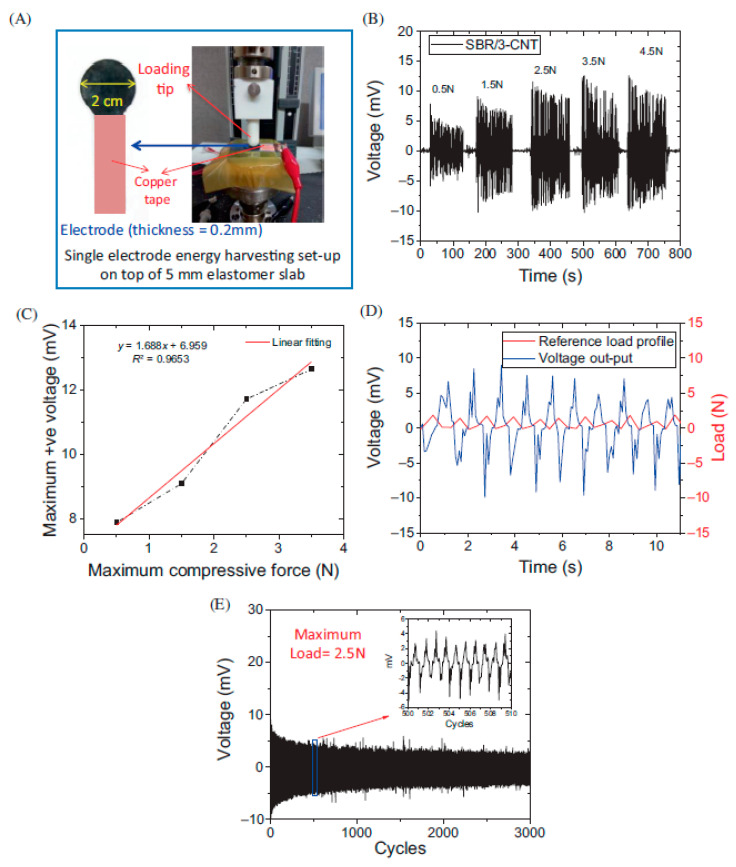
(**A**) Optical image of set-up. Loading tip was around 2 cm in diameter, and copper electrodes were attached to the flexible electrode for collecting the output voltage through a multimeter. (**B**,**C**) Output voltage under different forces. These reported results show that output voltage keeps increasing with increasing mechanical force up to 3.5 N before lowering down at 4.5 N force. (**D**) Output voltage under cyclic loading. (**E**) Durability examinations at a load of 2.5 N. Voltage was stable during the initial cycles but started to degrade after 500 cycles. This degradation is due to crack formation in the electrode and at filler–rubber interface failure. Reproduced with permission from Wiley [132].

In the present review, energy harvesting through piezoelectric principles is reported. The main mechanism of energy harvesting through the piezoelectric principle involves generating output voltage under the mechanical deformation of these composites. This involves the addition of piezoelectric materials in an elastomeric matrix [173]. The composites were then deformed under mechanical loads on the prototype used for energy generation. Then, the dipoles in the substrate containing these piezoelectric composites are polarized. Thereby, the further mechanical deformation helps further opposite change separation and finally migrated to electrodes through capacitance [174,175,176]. Finally, this charge can be harvested from the electrode, and the output voltage in the form of electrical energy can be obtained. Park et al. [176] report the setup for harvesting energy under different mechanical loads. Figure 9A reports the setup for obtaining output voltage through piezoelectric materials. The different magnitudes of mechanical loads range from 0.5 N to 4.5 N provided through a universal testing machine as shown in Figure 9A. Figure 9B reports the output voltage under different loads, and Figure 9C shows the maximum voltage generated under particular compressive force.

The relationship between the output voltage and mechanical force can be linear up to a certain mechanical force, like up to 3.5 N, or non-linear after a certain mechanical force like 4.5 N [176]. This change in output voltage under different mechanical forces depends critically on the type of materials used during fabrication, the composition of the ingredients, and the magnitude of mechanical force [177,178]. However, the composite material can exhibit piezoelectric or piezoresistive properties as per need. For example, they can generate an output electrical response under mechanical stress or strain as suggested by Kumar et al. [179]. Finally, Figure 9D shows the electro-mechanical behavior under cyclic mechanical force of 2.5 N and durability tests in Figure 9E. The results show that the output voltage was stable during the initial cycles, like in Figure 9D, but started degrading after ~500 cycles, as reported in Figure 9E in durability tests. There are various reasons for the degradation of the output voltage after 500 cycles. The main reason is crack formation in the composite electrode and substrate. These cracks can lead to changes in the material properties, such as reduced piezoelectric or piezoresistive coefficients like those reported by Manikkavel et al. [180].

**Figure 10:**
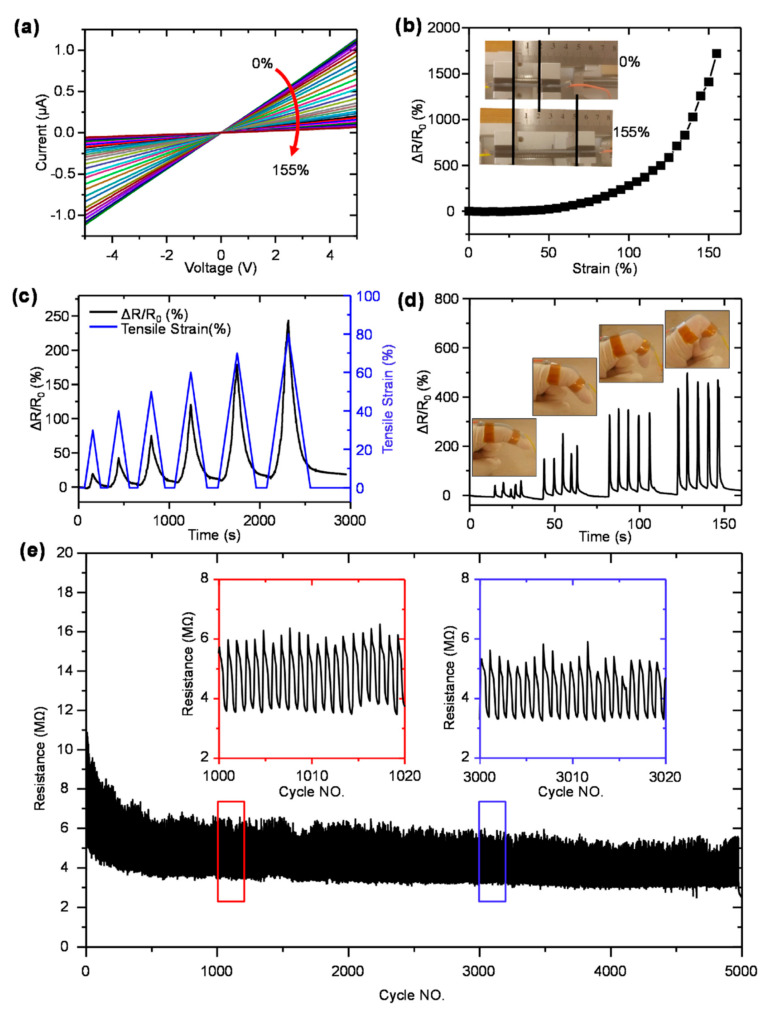
(**a**) Current–voltage (I-V) profiles of the samples under 0–155% strain. These I-V curves provide crucial insights into the nature of electrical behavior under change in mechanical loading. (**b**) Resistance change under uniaxial tensile strain. Results show that resistance to change increases to as high as 1719% at 155% strain. (**c**) Relative resistance change under tensile strain. (**d**) Real-time monitoring of sample by finger bending at different angles. These piezoresistive sensors can detect changes in shape or curvature as the finger is bent. (**e**) Durability cycle of sample. Study shows that adding MoS_2_ in hybrid with MWCNT improves fracture toughness and thereby excellent durability with negligible losses for up to 3000 cycles. Reproduced with permission from Elsevier [187].

This results finally results in a degraded output voltage. The fatigue leading to microstructural damage in mechanical or electrical components. Under repetitive loading-unloading cycles, the fatigue-related degradation can result in a decrease in the output voltage generated [181]. Here, the voids or fabrication defects in the composites and electrical contact degradations at the substrate-electrode interface region [182]. Therefore, proper materials with high fracture toughness must be selected to obtain high durability of the composites for obtaining promising performance and overcoming mechanical failure of the composites [183]. Thus, the role of improving fracture toughness in composites for obtaining good durability with minimum losses is described in Figure 10 in the coming section.

### 4.5. Current–Voltage Curves, Real-Time Monitoring, and Durability of Composites

It is well known that improving fracture toughness is crucial for obtaining good electro-mechanical stability and mechanical deformation. The high fracture toughness of the composite enables the composite material to resist crack development and propagation [184]. Such resistance to crack propagation helps in improving mechanical integrity under cyclic loading and thus good durability. Moreover, if materials with high fracture toughness are used, they will prevent mechanical failure under mechanical cyclic loading [185]. This will be especially useful for accurate and continuous monitoring of the composites for strain-sensing applications. Higher fracture toughness helps to ensure the accuracy and consistency of strain measurements over time [186]. This is because the mechanical degradation of the strain sensor is expected to be negligible, thereby maintaining the accuracy of the durability tests under constant cyclic loading.

Here, Alam et al. [187] report the piezoresistive behavior of the composites with higher fracture toughness in Figure 10. Figure 10a reports the behavior of the current–voltage (I–V) curve for the composite used in strain sensing. It can be understood as variations in electrical resistance or piezoresistance with a change in the magnitude of the applied strain [188]. These I-V curves depict the correlation between applied strain and resulting electric current in the composite sample. The other prospects in I-V curves involve their linear nature at small strain magnitude following Ohm’s law. However, at higher strains, the I-V nature is non-linear and leads to a change in slope and threshold voltage [189]. These I-V curves are useful as they can assist in estimating strain sensitivity, gauge factor, hysteresis, and linearity in strain-sensing applications. Figure 10b,c show the change in relative resistance concerning unidirectional and cyclic strain. Moreover, the relative resistance was higher at higher cyclic strains. This can be understood through an increase in inter-particle distance between electrically conductive fillers at higher tensile strain [190,191]. Moreover, as such piezoresistive materials exhibit an enhanced sensitivity to a higher magnitude of tensile strain. For example, as the material undergoes more significant stretching, its electrical resistance changes more noticeably at a given strain [192]. The main mechanism behind such effects involves the re-orientation of electrically conductive pathways within the composite material. This leads to a more pronounced change in relative resistance at higher levels of tensile strain [187].

The importance of fracture toughness was further validated by Alam et al. [187] by real-time monitoring of finger bending at different angles in Figure 10d. The main mechanism involves transferring mechanical deformation into relative resistance change with finger bending [193,194].

For example, as the finger bends, the sensor undergoes strain, causing changes in its electrical properties, such as resistance. The results show that the relative resistance changes with an increase in finger bending. Such changes can be studied and converted into meaningful data to represent the extent of finger bending. Finally, the durability of the strain sensor prototype is reported in Figure 10e. There are various aspects of improving durability by tuning mechanical stiffness. For example, the composite materials used in fabricating strain sensors are subjected to external mechanical load to obtain a stable piezoresistive response. However, it is subjected to mechanical failure after a certain number of cycles. Thus, Alam et al. [187] report the concept of improving fracture toughness to obtain stable and long durability with minimum voltage losses. For that, the MWCNT was added to the electrode in hybrid to MoS_2_ to obtain highly stable durability cycles [187]. The main mechanism behind this higher mechanical stability involves the reduction in the curing process of silicone rubber. It results in the development of uniform crosslink density and thus uniform and better mechanical properties [187]. The literature summary about the sensors and their properties are summarized in Table 1.

## 5. Conclusions

The present review article reports the prospects of carbon nanomaterials-reinforced silicone rubber composites. The use of these composites for industrial applications like physical activity monitoring sensors is reviewed for the years 2020 to 2025. The literature shows that the carbon-based material can be very promising for adding to silicone rubber during composite fabrications. For example, they improve the mechanical, electrical, and thermal properties of silicone rubber when used. These improved properties make these composites useful for industrial applications like sensors. The review provides further insight that the sensors studied through the literature survey show that these carbon materials not only make the composites based on them robust but also feasible for their multi-functionality. Moreover, silicone rubber acts as a great supporting rubber matrix because of its easy processing and electro-active properties. Finally, the literature survey shows that sensors based on silicone rubber and carbon materials exhibit high sensitivity, optimum relative resistance, high gauge factor, and, finally, robust durability. Therefore, the present review article allows the reader to understand the advantages of using these composites for various industrial uses as sensors or energy harvesting.

### 5.1. Advantages and Challenges

This review presents the robustness of the sensing technique and the role of filler, rubber matrix, and their properties in influencing the performance output. The review further insights that the use of frequently used carbon nanomaterials is novel for achieving high performance. These frequent materials are carbon black, carbon nanotubes, and graphene. Moreover, silicone rubber arises as the best rubber matrix to achieve such high performance. The advantages and challenges of these composites are discussed briefly below.

For carbon black prospects, there are various advantages to using carbon black, such as its cost-effectiveness and high surface area. Thus, it is traditionally used as a filler to reinforce the rubber matrix, as reported by Xu et al. [197]. These high mechanical properties make it useful in sensing with high performance. However, there are some challenges, such as the aggregation of carbon black particles when mixed with a silicone rubber matrix. Another challenge is its limited sensitivity, which leads to a poor gauge factor compared to carbon nanotubes and graphene, as reported by Song et al. [195]. Similarly, for the carbon nanotubes prospects, there are various advantages of using carbon nanotubes in sensing applications. They include high sensitivity and high gauge factors due to excellent electrical properties and high aspect ratio as reported by Pei et al. [198]. However, there are some challenges, such as achieving uniform dispersion. However, the functionalization of carbon nanotubes can be used to achieve good filler–rubber compatibility. Therefore, it helps to achieve uniform dispersion, but the process is challenging and involves acids and other hazardous materials like those presented by Peng et al. [199]. Moreover, it is relatively expensive compared to traditional fillers like carbon black. Finally, obtaining the proper orientation of carbon nanotubes is critical for optimum mechanical performance.

Then, for graphene prospects, the graphene exhibits various extraordinary mechanical and electrical properties, making it ideal for sensing applications. Its high reinforcing properties enhance durability and performance under mechanical strain, as suggested by Guo et al. [200]. However, there are many challenges, such as production and scalability, integration, and re-staking among the graphene layers due to van der Waals forces [201]. Therefore, new methods need to be developed to synthesize large-scale graphene production to exploit their outstanding properties and robust sensing. Silicone rubber is extensively used in sensing applications due to its excellent flexibility, biocompatibility, and high durability. It also serves as an excellent matrix material for fillers like carbon nanomaterials and creates robust sensors, as reported by Kumar et al. [196]. However, there are some challenges, like the dispersion of fillers, balance between conductivity and flexibility etc. Some other challenges are its low sensitivity, hysteresis losses, mechanical stability, and integration in sensing devices, as noted by Chen et al. [202]. Moreover, the medical silicone rubber grade exhibits toxicity that is added by the curing agents and fillers used in reinforcing the rubber matrix. Therefore, the right strategy must be followed to obtain balance properties like optimum stiffness, low toxicity, and high gauge factor or sensitivity for sensing applications, as indicated by Rahmani et al. [203].

### 5.2. Future Prospects of the Composites

The carbon nanomaterial-reinforced silicone rubber-based composites hold promise to revolutionize the sensing field. These applications include healthcare, environmental monitoring, structural health monitoring. Moreover, wearable technology is also promising perspective of these sensors, as suggested by Haghi et al. [204]. The unique mechanical and electrical properties of these composites provide a promising route for obtaining robust composites with high sensing capacity. For carbon nanotube prospects, carbon nanotube-based composites develop more sensitive and accurate wearable healthcare devices. These devices are useful for tracking vital signs such as heart rate and blood pressure [205]. Moreover, the advanced integrated system of fitness trackers can be useful to monitor physical activity, strain, and stress on muscles and joints. These composites are also useful for designing flexible electronics. For example, electronic skins can mimic human touch and pressure sensitivity for use in robotics, as reported by Palumbo et al. [206]. Finally, these composites are useful for environmental monitoring, including air and water quality. For example, the creation of more sensitive air and water quality sensors capable of detecting trace amounts of pollutants in air and water, as reported by Nasture et al. [207]. Graphene holds promising insights to develop high-performance sensors. These sensors include strain sensors, pressure sensors, chemical sensors, and biological sensors, as suggested by Li et al. [208]. In addition, graphene is promising to create self-powered sensing systems like energy harvesting or energy storage like batteries. The use of graphene also helps to achieve transparent and flexible electronics [209]. For example, the development of touchscreens, displays, and flexible circuits is available using graphene-based composites.

For carbon black prospects, carbon black holds promising solutions for cost-effective gadgets for sensing and reinforcing aspects of composites. For example, by using it in conductive rubber composites, flexible and stretchable sensors with improved mechanical properties can be created [210]. Moreover, carbon black can be useful in creating sensors for automotive applications, such as tire pressure monitoring and strain detection in vehicle components. Therefore, these composites are useful for multifunctional aspects in a wide range of applications, such as electronics, tires, and health monitoring sensing [211]. Silicone rubber reinforced with carbon nanomaterials also holds promising prospects. The first use of silicone rubber was in medical implants. This involves the development of biocompatible sensors for long-term monitoring of physiological parameters within the body, as reported by Kim et al. [212]. Moreover, they can be useful for wearable health monitoring. For example, it can achieve enhanced capability for real-time data collection and analysis for proactive health management based on silicone rubber. In addition, silicone rubber is also useful for environment sensing, structural health monitoring, and finally, robotics, as reported by Horne et al. [213]. Therefore, the composite reviewed in this paper has vast and promising prospects. The combination of these materials offers significant improvements in sensitivity, flexibility, durability, and biocompatibility, as reported by Han et al. [214]. These features articulate the way for advanced sensors in healthcare, environmental monitoring, and structural health monitoring.

### 5.3. Overview of Cost-Effectiveness, Biocompatibility, and Scalability for These Sensors

As discussed in this review paper, the composite-based sensors hold a promising front for high-performance sensors. These aspects are due to their flexibility, adaptability, and low mechanical modulus. However, their practical adoption requires overcoming factors such as cost-effectiveness, biocompatibility, and scalability. These aspects are crucial for commercial viability, particularly in fields like wearable electronics.

(a) **Cost-effectiveness**: For the sensors reviewed in this paper, a key advantage of rubber composite sensors is their potential for low-cost production. Generally, most of the materials, such as silicone rubber and polyurethane, are relatively inexpensive and abundant in nature. This makes the fabrication of these sensors at large scale feasible. Moreover, many commonly used conductive fillers (e.g., carbon black or graphite) offer a cost-effective option. These cheap fillers are alternatives to high-cost nanomaterials like graphene or metallic nanoparticles. However, cost challenges can arise when advanced fillers such as monolayer graphene or silver nanowires are used to achieve high performance. Another challenge is the use of complex fabrication techniques such as laser patterning and 3D printing. Overall, balancing performance with economic feasibility remains a central focus, especially for applications like single-use sensors.
(b) **Biocompatibility**: This review summarizes the importance of biocompatibility for the use of these sensors for wearable applications. For example, biocompatibility is essential for applications involving direct contact with human skin or implantation. Many rubber matrices, such as medical-grade silicone and thermoplastic polyurethanes (TPUs), are inherently biocompatible and useful with low-to-no toxicity. Moreover, they have a long history of use in biomedical devices. The choice of fillers, however, significantly affects biocompatibility. For example, the carbon-based fillers like CNTs and graphene in higher concentrations could make the composite toxic. Low content of fillers generally shows acceptable biocompatibility when embedded in a stable matrix. However, concerns remain about potential cytotoxicity if the fillers are released due to material degradation. To overcome this challenge, encapsulation layers and surface treatments are often employed to mitigate for long-term safety.
(c) **Scalability**: This review work gives insight into the prospects of scalability for these sensors. Here, the scalability is a major factor influencing the transition from research prototypes to market-ready products. Recently, simple processing techniques such as solution casting, screen printing, and roll-to-roll manufacturing have had a promising effect on scalability. For example, these techniques enabled the scalable production of rubber composite films and sensors. Therefore, these methods are well-suited for producing large-area or batch-fabricated devices. Nonetheless, challenges in maintaining uniform filler dispersion and ensuring integrating sensors into complex devices. These devices are useful as wearables or smart textiles and can limit scalability. Additionally, multi-step processes or post-fabrication treatments can increase complexity and reduce throughput.

### 5.4. Overview of Composite Sensors Compared to Alternatives Like Textile-Based or Capacitive Sensors

(a) **Composite sensor**: The present review describes the use of composite-based nano-invasive sensors and their use to monitor physical activities. These composite sensors are made by combining two or more different materials. These materials contain polymers and electrically conductive fillers like carbon nanotubes, graphene, or metal particles. This review paper further insight that these composites-based sensors can sense physical changes. These physical activities can be running, jumping, or walking via the piezoresistive principle. For example, the pressure, strain, and temperature originate from physical activities, and the sensor performs the change through changes in electrical properties. The key features of these sensors include high flexibility, light weight, tunable sensitivity, and multimodal sensing. For example, the tuning of the sensitivity of these sensors can be performed through filler content. Moreover, their capacity for multimodal sensing involves their sensitivity to changes in pressure, strain, or temperature. Finally, their light weight makes them useful for soft and wearable applications. However, there are some limitations to these sensors, like uniform filler dispersion, signal draft, or hysteresis losses, and finally, the durability is a great issue.
(b) **Textile-based sensors**: Textile sensors are greatly useful and made from conductive threads or coatings integrated into the fabrics. These sensors are also piezoresistive, where sensing is achieved through a change in resistance. The key features of these sensors are their seamless integration with garments, their light weight, and their ease of wearing. Due to these features, these sensors are comfortable to wear, scalable, washable, and washable. Moreover, they have high flexibility and are easy to fabricate. However, some challenges involve lower durability, higher sensitivity, and sensitivity to the environment. The sensitivity to the environment includes the change in temperature or humidity in the surrounding environment, which results in a change in resistance. Moreover, these sensors have limited multifunctionality and have less ability to detect multiple stimuli.
(c) **Capacitive sensors**: Capacitive sensors work by studying change in capacitance caused by mechanical deformations. These types of sensors are frequently used in touchscreens and pressure sensors. The key features of these sensors include fast response time, high sensitivity, and moderate flexibility. The advantages of using these sensors include high precision, non-contact sensing, and low power consumption. Due to their low power consumption, these sensors are frequently useful for military applications. However, these sensors have some limitations, such as sensitivity to humidity, limited flexibility, and sensitivity to external noise. Moreover, these sensors have limited multimodal sensing, moderate durability, and moderate-to-tough fabrication processes.

## Figures and Tables

**Table 1:** Literature summary on different aspects of composite-based sensors.

Materials Used During Fabrication *	Modulus	Elongation at Break	Electrical Properties	Response Time	Gauge Factor	Reference
MWCNT, SR	6.5 MPa	210%	0.5 kΩ	-	~1.4 at 125%	[18]
LIG, PI, PDMS	-	-	250 Ω	-	75 at 5%	[19]
NCB, PDMS				15 ms	20.97 at 35%	[27]
CNF, PDMS	-	-	10 kΩ/sq	~100 ms	11.11 at 50%	[46]
CNF, PDMS	-	-	-	~50 ms	417 at 100%	[47]
CNT, GNP, SR	11 MPa	-	0.7 kΩ	-	-	[60]
CNT, GNP, CB, SR	7.5 MPa	250%	-	-	7.8 at 100%	[64]
CB, f-GNP, SR	4 MPa	170%	5 kΩ	-	-	[87]
MWCNT, TiC, SR	3.87 MPa	210%	-	100–300 ms	23.9 at 22%	[91]
MWCNT, f-PDMS	-	-	-	12.88 ms	22.64 at 225%	[98]
CNS, TPU	-	200%	30 kΩ	-	57.94 at 100%	[107]
CFM, GO/PAN	-	50%	-	<300 ms	10,688 at 15%	[109]
PVA, Bn, PEI, MXene	-	-	-	120 ms	-	[111]
SnS_2_, PDMS	-	-	-	-	3933 at 1.2%	[112]
CNT, UHPC,	166.3 MPa	-	12.95 kΩ	-	28	[117]
MWCNT, Ecoflex	-	1300%	-	-	6.86 at 100%	[120]
Alumina, Kapton	-	120%	-	-	2.6 × 10^8^ at 70%	[128]
MWCNT, SBR	12 MPa	200%	-	-	48.62 at 187%	[132]
MWCNT, SR	3.5 MPa	220%	-	200 and 125 ms	3.7 at 40%	[136]
MWCNT, GNP, SR	9 MPa	175%	-		32.04 at 100%	[139]
PAA-PVA hydrogels	-	30%	-	-	2.03 at 15%	[141]
CIP, CB, PDMS		80%		96 and 50 ms	7.74 at 80%	[156]
MWCNT, MoS_2_, SR	0.9 MPa	325%			25.95 at 155%	[187]
SNW, TPU		360%			>16,000 at 360%	[190]
CCB, PVDF, ENR		60%	4 MΩ		1560 at 40%	[191]
CCB, Graphene, SR	7.7 MPa	200%			1.89 × 10^4^ at 44%	[195]
MWCNT, Thinner, SR	1.14 MPa	200%	-	540–110 ms	0.94 at 40%	[196]

* Abbreviations: MWCNT = multi-wall carbon nanotubes, SR = silicone rubber, LIG = laser-induced graphene, PI = polyimide, PDMS = polydimethylsiloxane, NCB = nano carbon black, CNF = carbon nano fibers, CNT = carbon nanotubes, GNP = graphene nanoplatelets, CB = carbon black, f-GNP = functionalized graphene nanoplatelets, TiC = titanium carbide, CNS = carbon nano structures, TPU = thermoplastic polyurethane, GO/PAN = graphene oxide polyanaline, PVA = polyvinyl alcohol, PEI = polyetherimide, UHPC = ultra-high-performance concrete, SBR = styrene butadiene rubber, CIP = carbonyl iron particles, MoS_2_ = molybdenum disulfide, CCB = conducting carbon black, PVDF = polyvinylidene fluoride, ENR = epoxied natural rubber.

## Data Availability

Not applicable.
